# Integrating Gestational Diabetes Screening and Care and Type 2 Diabetes Mellitus Prevention After GDM Into Community Based Primary Health Care in South Africa-Mixed Method Study

**DOI:** 10.5334/ijic.5600

**Published:** 2022-09-21

**Authors:** Jean Claude Mutabazi, Pascal Roland Enok Bonong, Helen Trottier, Lisa Jayne Ware, Shane Norris, Katherine Murphy, Naomi Levitt, Christina Zarowsky

**Affiliations:** 1 Département de Médecine sociale et Préventive, École de Santé Publique, Université de Montréal, Pavillon 7101, Avenue du Parc, Montréal, QC H3N 1X7, Canada; 2 Centre de Recherche en Santé Publique (CReSP), Université de Montréal et CIUSSS du Centre-Sud-de-l’Île-de-Montréal, Montréal, Canada; 3 Centre de Recherche du Centre Hospitalier Universitaire Sainte Justine, Montréal, H3T 1C5, QC, Canada; 4 SAMRC Developmental Pathways for Health Research Unit, Department of Paediatrics, School of Clinical Medicine, University of the Witwatersrand, ZA; 5 Division of Endocrinology, Department of Medicine, Faculty of Health Science, University of Cape Town, Chronic Disease Initiative for Africa, Cape town, Western Cape, South Africa; 6 School of Public Health, University of the Western Cape, Robert Sobukwe Rd, Bellville 7535, South Africa

**Keywords:** integration, primary health care, gestational diabetes, GDM, Type 2 diabetes, T2DM, South Africa

## Abstract

**Background:**

Despite high gestational diabetes mellitus (GDM) prevalence in South Africa (9.1% in 2018), its screening and management are not well integrated into routine primary health care and poorly linked to post-GDM prevention of type 2 diabetes mellitus (T2DM) in South Africa’s fragmented health system. This study explored women’s, health care providers’ and experts’ experiences and perspectives on current and potential integration of GDM screening and prevention of T2DM post-GDM within routine, community-based primary health care (PHC) services in South Africa.

**Methods:**

This study drew on the Behaviour Change Wheel (BCW) framework and used a mixed method, sequential exploratory design for data collection, analysis and interpretation. Individual semi-structured interviews were conducted with key informants (n = 5) from both national and provincial levels and health care providers (n = 18) in the public health system of the Western Cape Province. Additionally, focus group discussions (FGDs) with Community Health Workers (CHWs n = 15) working with clinics in the Western Cape province. A further four FGDs and brief individual exit interviews were conducted with women with GDM (n = 35) followed-up at a tertiary hospital: Groote Schuur Hospital (GSH). Data collection with women diagnosed and treated for GDM happened between March and August 2018.

Thematic analysis was the primary analytical method with some content analysis as appropriate. Statistical analysis of quantitative data from the 35 exit interview questionnaires was conducted, and correlation with qualitative variables assessed using Cramér’s V coefficient.

**Results:**

Shortage of trained staff, ill-equipped clinics, socio-economic barriers and lack of knowledge were the major reported barriers to successful integration of GDM screening and postnatal T2DM prevention. Only 43% of women reported receiving advice about all four recommendations to improve GDM and decrease T2DM risk (improve diet, reduce sugar intake, physical exercise and regularly take medication). All participants supported integrating services within routine, community-based PHC to universally screen for GDM and to prevent or delay development of T2DM after GDM.

**Conclusion:**

GDM screening and post-GDM prevention of T2DM are poorly integrated into PHC services in South Africa. Integration is desired by stakeholders (patients and providers) and may be feasible if PHC resource, training constraints and women’s socio-economic barriers are addressed.

## Background

Between 10% and 31% of type 2 diabetes (T2DM) cases among women are reported to be associated with previous gestational diabetes mellitus (GDM), and the risk of developing T2DM is increased more than 7 fold for women who had GDM compared to those without [1,2]. The national prevalence of gestational diabetes (GDM) in South Africa was estimated at 9.1% in a 2018 study [1]. The authors of the study warned of subsequent Type 2 diabetes (T2DM) for these women and their children along with complications, reduced longevity and impacts on the national health system [3].

The first step towards optimal management of GDM and prevention or delay of subsequent T2DM is diagnosis. GDM screening for all pregnant women has therefore been recommended by several professional bodies [4,5]. Currently, only a minority of women get screened worldwide for GDM [5], using many GDM testing and diagnostic criteria that have not been standardised despite efforts to do so [4] (See Table 1 for different GDM diagnostic criteria).

**Table 1:** Different diagnostic criteria for GDM.


DIFFERENT DIAGNOSTIC CRITERIA TO DIAGNOSE GDM		

GROUP/ORGANISATION	SCREENING TEST	DIAGNOSTIC CRITERIA: BLOOD GLUCOSE LEVEL THRESHOLDS

American Diabetes Association [6,7]	One step: 2 hr 75 g OGTT	At least one of the following must be met: Fasting: ≥5.1 mmol/l (92 mg/dl) 1 hr: ≥10.0 mmol/l (180 mg/dl) 2 hr: ≥8.5 mmol/l (153 mg/dl)

	OR Two step: 1) 1 hr 50 g (non-fasting) screen 2) 3 hr 100 g OGTT	OR If 1 hr: ≥10.0 mmol/l (180 mg/dl) proceed with step 2 3 hr: ≥7.8 mmol/l (140 mg/dl)

Carpenter and Coustan [8]	3 hr 100 g OGTT	At least two of the following must be met: Fasting: ≥5.3 mmol/l (95.4 mg/dl) 1 hr: ≥10.0 mmol/l (180 mg/dl) 2 hr: ≥8.6 mmol/l (154.8 mg/dl) 3 hr: ≥7.8 mmol/l (140 mg/dl)

Diabetes Pregnancy Study Group (DPSG) of the European Association for the Study of Diabetes (EASD) [9]	2 hr 75 g OGTT	Fasting: >5.2 mmol/l (93.6 mg/dl) OR 2 hr: >9.0 mmol/l (162 mg/dl)

International Association of Diabetes and Pregnancy Study Groups (IADPSG) [10]	2 hr 75 g OGTT	At least one of the following must be met: Fasting: ≥5.1 mmol/l (92 mg/dl) 1 hr: ≥10.0 mmol/l (180 mg/dl) 2 hr: ≥8.5 mmol/l (153 mg/dl)

National Diabetes Data Group (NDDG) (1979) [11]	3 hr 100 g OGTT	At least two of the following must be met: Fasting: ≥5.8 mmol/l (105 mg/dl) 1 hr: ≥10.6 mmol/l (190 mg/dl) 2 hr: ≥9.2 mmol/l (165 mg/dl) 3 hr: ≥8.0 mmol/l (145 mg/dl)

World Health Organisation (1985) [12]	2 hr 75 g OGTT	Fasting: ≥7.8 mmol/l (140 mg/dl) OR 2 hr: ≥7.8 mmol/l (140 mg/dl)

World Health Organisation (1999) [13]	2 hr 75 g OGTT	Fasting: ≥7.0 mmol/l (126 mg/dl) OR 2 hr: ≥7.8 mmol/l (140 mg/dl)

World Health Organisation (2013) [14]	2 hr 75 g OGTT	At least one of the following must be met: Fasting: 5.1–6.9 mmol/l (92–125 mg/dl) 1 hr: ≥10.0 mmol/l (180 mg/dl) 2 hr: 8.5–11.0 mmol/l (153–199 mg/dl)


While necessary, screening and diagnosis alone are insufficient. Better follow up of women with GDM in order to reduce the risks of developing T2DM requires better coordination between antenatal and postnatal care [15,16]. Ideally, this could be achieved through integrated services for all conditions, or which tackle specific diseases and populations – notably the post-partum care of women’s obstetric and other health care needs, and the care of infants and children. Therefore, such strategy would be feasible with an approach integrating prevention, diagnosis, treatment, and palliative care for all conditions that could be managed within PHC [17].

In South Africa, women are screened for GDM based on risk factors, as one element of ante-natal care (ANC) [18,19,20]. Women diagnosed with GDM are then referred to tertiary hospitals for their pregnancy follow-up and delivery, but only a small proportion of these women return for postpartum assessment, including an oral glucose tolerance test (OGTT), and management [21,22]. This gap between antenatal care and postnatal follow-up is being investigated in high income settings where many women report intentions to change their lifestyle post GDM to prevent T2DM onset, even though they find it challenging [15,23]. There is little evidence from low and middle-income countries like South Africa on actual implementation of guidelines, nor of the feasibility and acceptability of potential strategies to improve continuity and integration of care for women who have had GDM [24].

In contrast to the very low levels of routine post-partum glucose assessment following a GDM pregnancy [15], women in South Africa routinely bring their newborns and infants to clinics for immunization and well-baby care [25,26]. Integrating GDM care and prevention of T2DM post GDM within primary health care (PHC) in South Africa would facilitate women’s access to services in one place. This would decrease the burden of navigating a fragmented health system for their own care and the care of their babies. In recognition of this fragmentation of care in South Africa, experts have called for integrated health systems and services that are easy for patients to navigate [27].

This study explored women’s perspectives and knowledge of their own GDM and post-partum care, as well as the perspectives of health care providers, in order to assess both the current degree of integration of GDM screening and prevention of T2DM post-GDM within routine, community-based primary health care (PHC) services in South Africa. The perceived acceptability and feasibility of greater integration of these critical aspects of women’s health care to these key stakeholders were also explored.

## Methods

### Study framework

**Figure 1:**
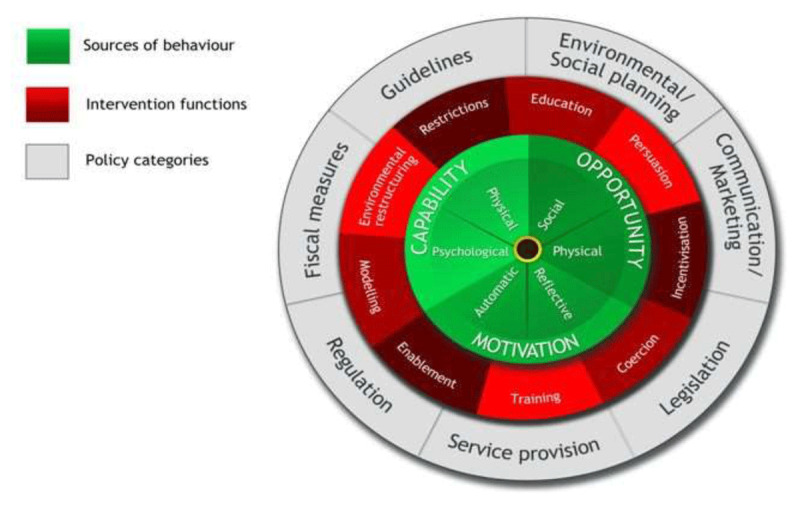
Behavioural Change Wheel framework [28] (Figure used with permission of Prof. Susan Michie).

The Behaviour Change Wheel (BCW) framework [28] (Figure 1), was used for this study. BCW has been effectively applied to planning and evaluating interventions targeting individuals, groups, programmes and behaviours [29,30]. While motivation, capability and opportunity from the model’s inner circle are seen as the enablers for both individual and collective behaviour change, they must be assessed in relation to programme implementation, its mechanism and context [31,32]. In this study, the BCW was used to understand the *policies, practices and barriers to change* among health workers and women.

### Study design and settings

A mixed method, sequential exploratory design was used, applying the Mixed Methods Appraisal Tool (MMAT), version 2018 [33,34].

This study contributes to the process evaluation of an ongoing complex intervention implementation research project, IINDIAGO (an Integrated health system Intervention aimed at reducing type 2 Diabetes risk in women after Gestational diabetes in South Africa, Trial ID: PACTR201805003336174), which aims to integrate improved post-partum follow up for women with GDM into PHC and thus contribute to T2DM prevention in two South African cities: Cape Town, Western Cape province and Soweto township in Johannesburg, Gauteng province). The IINDIAGO trial was in the recruitment stage among women from disadvantaged communities in Cape Town and Soweto, South Africa, when data were collected for this nested study. Data collection was conducted among women who attended Groote Schuur Hospital (GSH) and health care providers in health care facilities within the public health care system in the Western Cape province. Western Cape serves its population primarily through a network of clinics providing PHC services and serving as the entry point into the health care system, including two central, tertiary university teaching hospitals, an obstetrics referral hospital and one specialized pediatric hospital (Tygerberg Hospital, GSH, Mowbray Maternity Hospital and Red Cross War Memorial Children’s Hospital) for specialist services [35]. PHC services in the Western Cape province are managed by two separate health authorities, Municipal City Health (in the Cape Metro Health District) and provincial Department of Health (DoH). Most district facilities are managed by the provincial DoH. The exception is Cape Metro Health District, which is managed by both City Health and provincial DoH. The Western Cape Province has 479 public PHC centres and these include clinics, of which some mobile and satellite clinics are under the authority of City Health. The provincial DoH manages Community Day Centres and Community Health Centres. All these clinics refer patients to the district provincially aided, regional, specialist and tertiary hospitals available in different parts of the province [35]. Health care providers who participated in the study were recruited from some of these clinics.

### Data sources and eligibility criteria

The data sources included: Firstly, semi-structured in-depth qualitative interviews with key informants (KIs) (N = 5) and health care providers (HCPs) (N = 18). Secondly, focus group discussions (FGDs) with women diagnosed with GDM (N = 35 women in 4 FGDs) and community health workers (CHWs) (N = 15 CHWs in 2 FGDs). Additionally, exit interview questionnaires with the 35 women who participated in FGDs for further exploration of associations between qualitative variables.

The KIs included researchers, policy makers and clinicians who have been involved in DM policies and care, especially for GDM and T2DM, at national and provincial levels. They were interviewed about GDM policies and their experiences and perspectives on integrated GDM screening and T2DM prevention within PHC services. HCPs included managers, nurses or midwives from the public health sector in Cape Town (WC province), South Africa. They were interviewed about their practices or processes for GDM screening and care in facilities, including referral pathways. Drawn from these same local facilities, CHWs were recruited to FGDs to share their experiences and roles working with patients including those who had GDM and T2DM. Women with GDM referred by different clinics in Cape Town and attending GSH for their GDM follow-up and delivery, were identified from hospital records, contacted and recruited to participate in the FGDs and share their GDM knowledge and the challenges they face while seeking care. These women also completed the brief exit interviews using questionnaires, regarding their background and on how their own GDM is being managed. These datasets were used to measure the correlation between qualitative variables. All research participants discussed their views of whether and how integrated health services such as those proposed in the IINDIAGO trial could help with GDM screening and initiatives for T2DM prevention among women in SA.

Participants aged more than 18 years without any cognitive disabilities were included in this study. All were able to communicate in English. In cases of women with GDM and CHWs who did not speak English well, participants were encouraged to express themselves in isiXhosa or Afrikaans and their responses were contemporaneously translated into English by the research assistant who was fluent in these local languages. The KIs and HCPs were offered no compensation upon completion of interviews. Women with GDM who participated in this study were provided with a R100 ZAR (around $7 USD) voucher while CHWs shared refreshments after FGDs. Fieldwork and data collection were conducted between March, 2018 and August, 2018.

### Study sample and data collection

Beginning with two experts recommended by the IINDIAGO principal investigators, sequential referral snowball sampling [36] was used to identify and recruit other KIs, who were then approached and recruited for this study. Criterion sampling [37,38] was used to identify all other respondents depending on their occupations or their GDM diagnosis and referral to GSH. Managers and nurses or midwives involved in GDM screening at the clinics, CHWs who (in coordination with the local facilities) deliver services to women with different health problems in the community and assist at the clinics when called upon, were selected using this sampling strategy.

Interview/FGD guides and exit interview questionnaires were respectively used as tools to collect qualitative and quantitative data. KIs were interviewed in their offices at the hospital, clinic or research facilities. The two FGDs conducted with CHWs were organised in collaboration with the two local clinics with which they were affiliated. The four patient FGDs included 6-10 women diagnosed with GDM and receiving care at GSH (N = 35), followed by individual exit interview questionnaires that took place in a room provided by the maternity ward at GSH. All interviews and FGDs were conducted by a trained researcher (JCM), assisted by a trained research assistant (SK) fluent in local languages, under the supervision of experienced qualitative researchers (KM and CZ). The researcher (JCM) introduced himself as a doctoral student and briefly interacted with the participants about the study before commencing the interviews and FGDs. Interviews with HCPs and FGDs with CHWs took place at the clinics, in their clinic offices for HCPs and in the rooms provided by the local clinics for CHWs. Each interview lasted between 30 and 45 minutes. FGDs lasted between 45 minutes and 1 hour. The Exit interviews lasted between 10 and 15 minutes. All interviews and FGDs were audio recorded and ATLAS.ti software was used to assist data analysis and management.

### Data analysis

The interviews and FGDs were transcribed and a coding system was developed by JCM in collaboration with CZ using an inductive/deductive approach. All discrepancies in the coding process were discussed and resolved between these two investigators. Thematic analysis was generally used but content analysis was applied on a few occasions in order to check the frequency of important codes [39,40]. For statistical analysis of the 35 exit interviews questionnaires, categorical variables were summarized using absolute frequencies and relative frequencies. Continuous variables were synthesized using central trend statistics (mean, median) and dispersion statistics (standard deviation (SD), interquartile range (IQR)). Qualitative variables were four advices for women (improve diet, reduce sugar intake, physical exercise and regularly take prescribed medication) to improve their GDM and prevent T2DM and Nurse’s concerns about health of these women. The correlation between these qualitative variables measured using Cramér’s V coefficient which is interpreted as follows: from 0.0 to <0.1 negligible association, from ≥0.1 to <0.3 weak association, from ≥0.3 to <0.5 moderate association and ≥0.5 strong association [41].

This analysis has also contributed to the ongoing process evaluation of the IINDIAGO study.

### Ethical approval

Ethical approval was obtained from the Human Research Ethics Committee, Faculty of Health Sciences, University of Cape Town (HREC REF: 946/2014), the City Health Department, Cape Town and the Department of Health, Western Cape, South Africa; and comité d’éthique de la recherche en sciences et en santé (CERSES), Université de Montréal (CERSES-19-058-D), Canada. Written consent was given for all interviews and the anonymity of participants was maintained throughout the research process.

## Results

In total, 73 individuals participated in this study. Participants in the in-depth individual interviews (N = 23), included 4 (17%) clinic managers and 14 (61%) nurses and midwives and 5 expert KIs (22%). Of these 23 respondents, 19 (83%) were female, with a mean age (SD) of 42.7 (SD 10.6) years and 16.1 (SD 11.0) years of experience in health care (see Table 2). Participants in FGDs (N = 50) included women with GDM and CHWs and were all female.

**Table 2:** Characteristics of KIs and HCPs.


FACTOR	LEVEL	VALUE

N		23

Gender	F	19 (83%)

	M	4 (17%)

Age (in years)	mean (SD)	42.7 (10.6)

	median (IQR)	41.0 (35.0, 47.0)

Experience (in years)	mean (SD)	16.1 (11.0)

	median (IQR)	12.0 (7.0, 23.0)

Category	Clinic managers	4 (17%)

	Nurses and midwives	14 (61%)

	KIs	5 (22%)


The four thematic categories that emerged from the analysed data were interpreted using three BCW layers from outer to inner: policy categories, intervention functions and sources of behaviour respectively. Each category was linked to a specific layer except the third and the fourth categories that were classified using the same “inner” layer (See Table 3).

**Table 3:** Categories and BCW layers.


CATEGORY	BCW LAYER AND MAIN CONTENT, FROM OUTER TO INNER

I. Existing guidelines, services and current practices in the clinics	Outer layer: policy categories

II. Effective antenatal referral procedures but lack of follow-up after delivery	Middle layer: intervention functions

III. IINDIAGO, an intervention with potential to bridge the gaps	Inner layer: sources of behaviour

IV. Encouraged role of CHWs involvement toward community based T2DM prevention intervention	Inner layer: sources of behaviour


Each category had different themes with each illustrated by a single quote from one of the participant groups. More illustrative quotes from various participants are depicted in Table 4.

**Table 4:** Categories and illustrative quotes.


KEY FINDINGS AND ILLUSTRATIVE QUOTES

Category 1: **Existing guidelines, services and current practices in the clinics** **Current GDM screening/care guidelines and its poor implementation** *“So, what we basically do in our facility, so we go according to the BANC protocol. We have our own protocol. If a mother comes in the morning for an antenatal booking, then we test her urine…”*. **HCP 1**. **From no testing to the risk-based screening of GDM at the clinics** **Risk factors assessment** *“There are two Community Centres in Gugulethu, the, and then it’s us, the mobile Baby Clinic. In our clinic it’s basic antenatal care, so the people who have a history with parents who are diabetic, usually we send them to the MOU, they are screened that side. We don’t do screening in our clinic. We don’t actually do that”*. **HCP 3**. “*Not every mum, but if she presents risk factors such as a family history of diabetes, the mum had a previous history with Gestational Diabetes, she has an exceeding Body Mass Index (BMI) and then if we tested the random blood sugar and found that it was above 7.8, then we will give the mother a Gestational Diabetes check…”*. **HCP 4**. *“So we do a random blood glucose at the facility, and depending on that result, we will then follow the necessary steps. There is obviously a screening in terms of family history, and have you had Diabetes before, or do you currently have Diabetes….”*. **KI 6**. **Process of GDM testing and referring women with GDM** *“the procedure for screening, we’ve got a list of indications for doing Glucose Tolerance Test (GTT): family history of diabetes from her mother, her father or her siblings, BMI of 35 and above, history of big babies, persistent Glycosuria; for three consecutive visits. She has to come in the morning, fasting, her last meal the previous night around 10 o’clock. So, when she comes, we do the prick. If the sugar is 7 and above, we don’t continue, but if it is less than 7, we take the fasting blood and we give her 75 grams of glucose, and we take the second blood after two hours. So, they come after one week for the results. If it’s an IGT, we refer to Mowbray not Groote Schuur, but if it’s GDM, then we refer to Groote Schuur”*. **HCP 2**. *“when they come here for the first time, we do the IGT (Impaired Glucose Tolerance) test or sugar test, and then if there is family history like the mother was diabetic, then we do the OGTT test, which is the fasting glucose, but we don’t do it here. I have to book for them in Gugulethu, and then they are going to give me the date when the patient can go there. Otherwise we have the forms that we use. We just take… I’m going to show you later the forms, and then we take, if the patient has already diabetes and she does not qualify to book here at the clinic, so I refer the patient straight to Gugulethu MOU”*. **HCP 5**. **Barriers to GDM screening into PHC** *“Well, the current practice is to try and identify them from women who attend antenatal care. That obviously means, the people who don’t attend, we wouldn’t pick it up…”* **KI 2**. *“You know, unfortunately a lot of the patients are picked up a bit later. The patients we pick up earlier of course, are those who previously diabetes, which is a different ballgame. So those get to come in early, but the majority of the patients come in at a later time…”*. **KI 3**. *“The only challenge is that when you give an appointment for the lady to come to do bloods, then she doesn’t come. Then it will be picked up because they are supposed to do it before they are 28 weeks; or if you do it at 28weeks then you have to repeat it. If it was borderline then you have to repeat, so then you don’t have that chance of checking if you pricked them already at seven months or close to eight months, so you don’t have that chance of checking, then you are going to refer them, because they are already late in pregnancy”*. **HCP 6**. *“….The presentations are varied, and 50% of patients that are currently diabetic don’t know yet that they have Diabetes. So I think anyone allied to the healthcare should be thinking about screening and actually being able to screen….”*. **KI 1**. *“I don’t think it’s okay, because sometimes we miss them, because maybe, it depends, maybe the family doesn’t have diabetes and the person can develop Diabetes during pregnancy. So sometimes, if it’s not picked up in the urine, and we don’t often do the diabetes test every time, it’s not like Hypertension, it’s not… I don’t think we are doing a good job in this case. There are no signs you know, if it’s high….”*. **HCP 2**. *“We don’t have time to talk individually, but at times when we give the Health Talk, we do explain to them…”*. **HCP 4**. *“You call an ambulance to pick up the clients to take to the MOU, or Mowbray, depending on where the pathway is. Now we send the letter. On the letter there is a sleeve that is supposed to come back to us, but that has never happened. I have been here for more than eight years now, I have never seen that sleeve coming back…”*. **HCP 7**. *“She must bring her own food, because we do not have glucose to eat. She goes and has breakfast, and then two hours later we re-prick…”*. **HCP 1**.

Category 2: **Effective antenatal referral procedures but lack of follow-up after delivery** **On-site integrated hospital services** *“she gets referred to Groote Schuur Hospital’s antenatal clinic where they will do what we call OPD (Out-Patients Department) spreads, and then they will start treatment; but the first line treatment for any diabetic is diet, and so she will see the dietician, lifestyle changes, and then she will start treatment…”*. **HCP 8**. *“nurses play an indispensable role in managing these patients, bearing in mind that the maternal and foetal wellbeing will be first assessed by nurses, and also nurse will also help in providing anthropometric measurements, they help to also reduce the time-lapse in some of these patients to spend a very long time waiting for doctors. So basically, nurses play a role in monitoring of the mother and the baby, as well as even sometimes in diagnosis and also in management”*. **KI 5**. *We all have our specialities, so the registrar that would be looking after the patient is somebody that is rotated through the whole block, so they’ve seen cardiac, they’ve seen eclamptic patients, they’ve done diabetes; but if there is a specific problem, then we are in the fortunate position where we have the resources where we can get infectious disease people out, instead of struggling with that, or we can get the endocrinologist out, and say listen, we have now hit a wall, how do we go forward, but that is within our setting”*. **HCP 8**. **Socio-economic boundaries to healthy antenatal and postnatal initiatives** *“…. sometimes when you check in, they say you must come without eating to the clinic and then they take a long time to check your sugars, and then you get tired, you are hungry. You know how you are when you’re hungry, you seriously want to”*. **Participant in FGD 1**. “*…. Like delays, and it’s now the strike, so there are no busses, so the trains are full; taxis you have to wait in line, and you know when you are pregnant, to stand for a long time in line, it also causes back pains; like now, I’ve got a huge back pain from standing in the line”*. **Participant in FGD 4**. *“…. Sometimes you just want to ask a small question and the sister goes to levels like (she gets upset and shout). She doesn’t even know the question that you want”*. **Participant in FGD 1**. *“…You come from work, even if you get your day off, you are tired, thinking about exercising, even if you want to, but your body doesn’t allow you to do so, because you are tired”*. **Participant in FGD 3**. *“…For me it’s very tough, to change my diet, because I’m used to eating. For me it’s really… and it’s not easy; that is why I’m cheating sometimes*. **Participant in FGD 2**. **Confusion or little knowledge of women on GDM and lifestyle changes** *“…this is my second child, I didn’t have sugar. Nobody in my family has sugar. I find out my sugar is high in my blood, so the doctor explained to me I must go on the Insulin, because otherwise I can have a miscarriage; and I don’t understand actually, maybe can it be, or what….”*. **Participant in FGD 2**. **Poor communication and inexistent plans for postnatal follow-up** *We don’t have a six week visit. We don’t have a six week visit. When six weeks postnatally, the baby is six weeks, so at six weeks they go to the Baby Clinic, so they don’t come back to us, that’s the thing*. **HCP 9**. *“There is no strict channel. Obviously, there is very detailed discharge information about what the diagnosis is, what the implications are, and what needs to be done in the interim. But as to whether people phone and follow up…? You know, there isn’t that, and there needs to be; not only in the management of GDM, but in the management of a lot of patients that we see for whatever medical reason…”*. **KI 3**. *“So, I think firstly the doctors and nurses don’t always have enough time, and also, they’re not very knowledgeable, and then even the dieticians are sometimes giving the wrong messages because of this whole debate internationally. So, I think those things are a problem, and then there is also the issue of healthy foods being expensive in townships, and the issue of exercise is difficult. I mean, if women get up at five o’clock, go to work in the town, go back, don’t get home till seven, you know, their lifestyles aren’t conducive to exercise”*. **KI 4**. *“The maternity sisters do not communicate with the local clinic sister for follow-up on these clients about medication after delivery and then we don’t know. So, maybe they got letters from hospital that you must follow up at this clinic to get your medication that is going to control you but mothers don’t follow up, as I have noted, they don’t follow up, they only focus on the baby after delivery, they focus on the baby. They don’t go for that follow up appointment and the medication, especially after they are coming from Maternity. But if there is a problem, then the doctor prescribes when discharging them but they will never mention it to us at the clinic…And then, if they are with the person who didn’t see them when pregnant, you won’t know if the client had a problem with the glucose”*. **HCP 10**. *“I mentioned earlier about the six weeks postnatal visit that needs to take place, and our nurses are overworked and understaffed, and I can attest to that. On any given day it is hectic in front, and staff shortages and absenteeism and people not adequately trained. People get pulled from one department to another to go and help out, and so all in all, what I’m trying to say is the six weeks postnatal visit, I don’t think to my knowledge that it is actually happening, that is, not in our facility”*. **KI 6**.

Category 3: **IINDIAGO, an intervention with potential to bridge the gaps** *“It is now policy. We had it two or three years ago, we wrote a postnatal care policy for the Western Cape, and I was involved in writing it, and it’s agreed, it’s just no-one has implemented it. So, it has to be implemented.… So, I do think it needs to be resourced. You need another nurse, and you need a particular training to give that nurse the referral route. So, what does she do with a person who’s depressed at six weeks? What does she do with the one who had GDM and they’ve checked her sugar now and it’s normal? What do they do with her? So, I think it needs almost a little bit of a syllabus for what the nurse does, you know?”*. **KI 4**. *We see it with IGT patients who are very well counselled and can actually reverse the whole and become normal. So, I think it’s feasible. I think it’s good that it (IINDIAGO) will give you raw data that you can then present to policymakers and say, listen, although we knew this, this is the hard data, done in a methodologically robust manner, and that no-one can argue with. And once faced with that, then one will have to change policies, and be forced to change the infrastructure and the way the infrastructure is set up to deal, not only with Type 2 Diabetes but with many other problems*. **KI 3**. *“I think if we can implement it (IINDIAGO) at the Well Baby Clinics for instance if they have enough staff and they are well-trained, I think it would make a big difference, because as a mother sometimes you are more worried about your baby, so then you are more likely to access that service; and then I think, like I said earlier, a continuation of care is better… So, if she has that continuous support at the Well Baby Clinic, because that is a place where she will be accessing the services quit e frequently, so she will be able to build a bond or a type of relationship with that caregiver on that side as well”*. **HCP 8**. *“It (IINDIAGO) is a good thing, because we such type of intervention we will normally check if everything is good when they come for post-delivery. We now just focus on breastfeeding and not in that side. We don’t go on the Diabetes side and Hypertension and all those things”*. **HCP 6**.

Category 4: **Encouraged role of CHWs involvement toward community based T2DM prevention intervention** *“You see, I think because the women with GDM after pregnancy, most of them don’t have any medical problem, the doctor or nurse will think they are wasting their time at the clinic, so, actually the initiative should be a community based one through lifestyle, and I think the community health workers are most important…but I don’t know the answers about the scope of a community health worker”*. **KI 4**. *“We also have health care workers that are not based in the clinic, but they report. Those are the people that are helping us work or supervising the ART or TB treatment for the people that are placed in the community to take their treatment. They visit. Even with the immunisation that is really not doing so well, they are able to the visits, the home visits. They are in contact with the community, so they also can help in this intervention (IINDIAGO)”*. **HCP 10**. *“…they also help us with recalling the mothers for other things. I think they can also play a role in this intervention (IINDIAGO)”*. **HCP 11**. *“For me, I think that the community workers are people from the community, so, the patients trust them more than coming to a sister in a hospital they can only see once.… so, the community knows them. If they do the screens and stuff they tend to trust them more than us some times. Yes, I think they need to be trained, because the last time I said they even need to be trained in doing prognostics for us, then they can do the diabetic screening at the same time, different screenings; because they are there in the house with ten people around them, so they can do all of that, and then they catch them early, even the blood finger prick”*. **HCP 4**. *“We have this form called household chart, here is a copy. So, inside house with the members of the household, and then you ask all these questions. Maybe there is someone who has symptoms of TB, who is HIV Positive, who is interested to test, then you advise to go and test. I give some card that we write in for follow-up on that date, referral cards. You tell them go to Crossroads if you are feeling that you are hypertensive and get your medication there. Diabetics, they talk a lot like I am drinking a lot of water. I am always tired, they talk about all those symptoms to you when you get there, so you record them and you check all the symptoms…You advise them about immunisation, Vitamin A, etc”*. **Participant in 1, FGD 1**. *“And even if it’s difficult, and they don’t want to come to the clinic, you as a CHW, you help her to start medication again. You educate people about their health, you tell them what is going to happen to them if they keep doing this or that? For example, you say to the patient that if you don’t go there and take your medicine, this is going to happen to you”*. **Participant 1, FGD 2**. *“Me, I love the job that I am doing because I don’t have a problem with people, and I can convince them but if someone is not doing well, I report her to the supervisor who will then intervene”*. **Participant 3, FGD 1**. *“My challenge is work load. We have to record. We have to be ready to give weekly and monthly statistics for our work. It’s a challenge, because there is a lot of work. We have to visit the clients, rain or shine, you have to visit them. You must have the minimum six to eight, and then each and every day you must have something to write down as proof of what you have done for the day. We must also cover many households at a long distance and reach target…*. **Participant 5, FGD 1**. *“Sometimes when we arrive at a patient, we see a number of men smoking. The whole house is like snow, so I am afraid of entering that house fearing what could happen to me when I enter that house”*. **Participant 3, FGD 2**. *“Challenges also include robberies in the community and even here at the clinic, they just come and attack you at the clinic’s gate and sometimes we are not working with our cell phones because we are afraid of robbery by the gangsters. And sometimes, even in the houses that they are going to do the pill counts in, they mustn’t go alone. We must therefore be two or three but it is not easy to get that one to make a friend and go together to avoid those incidents”*. **Participant 4, FGD 2**.


### The existing guidelines, services and current practices in the clinics

The BCW’s policy categories or outer layer [31] was used to assess the process of policy development, analyse its implementation and interpret insights from the KIs and the HCPs regarding the existing guidelines, services and current practices in the clinics regarding GDM screening, care and post-partum T2DM prevention initiatives. Perspectives and experiences of CHWs and women who participated in FGDs mostly referred to services they received and the practices in the clinics they attended. The results for this thematic category were subsequently grouped into 3 headings: 1. Current GDM screening/care guidelines and their implementation; 2. From no testing to risk-based screening of GDM; and 3. Barriers to GDM screening in PHC.

## Current GDM screening/care guidelines and their implementation

Both National and Western Cape departments of health introduced guidelines developed by experts based on international protocols to screen, diagnose and treat GDM [21], in all public health facilities. However, challenges arise in the implementation of these guidelines at local facilities. While discussing how GDM is diagnosed within ANC, KIs and HCPs reported that GDM screening guidelines have been poorly implemented at primary care level, resulting in missing some women with potential GDM.

*“We have screening protocols, and the South African Endocrinology Society has put out screening guidelines* [*for GDM*]. *Unfortunately, I think our screening is poor. We don’t screen widely enough, and there are many risk factors that aren’t screened….”*. **KI1**

Another implementation issue raised by participants related to counselling sessions regarding lifestyle changes to deal with diabetes and its devastating consequences. This included existing group counselling in ANC clinics and the individual and group sessions conducted through the IINDIAGO trial. Crowded clinics and inadequately equipped staff in some health settings were not conducive to effective group sessions and made individual counselling sessions almost impossible.

## From no testing to risk-based screening of GDM

### Risk factors assessment

Based on the current guidelines, GDM screening was supposed to be included in all ANC services and uniformly conducted in all local facilities. However, clinics approach GDM screening for pregnant women in different ways; some only test urine and then refer those with glycosuria, while others conduct confirmatory blood glucose tests before referring women to the next level of care. HCPs emphasised that screening decisions depend on the HCP’s assessment of risk factors that women present with during their ANC visits. Thus, not all women who attend ANC are tested for GDM in all clinics despite the ANC guidelines.

*“Firstly, I think I should explain that we are doing basic antenatal care. We then are taking care of women who don’t have any high risks, or just a normal pregnancy. If one is found to have sugar that is, glucose actually, that is evident in the urine, then we refer them, because we don’t even do them, the fasting stuff, so we will refer them to Gugulethu MOU, that’s where all the screening gets done. So, we take care of just the normal without any risk antenatal patients”*. **HCP 1**.

### Process of GDM screening and referring women diagnosed with GDM

**Figure 2:**
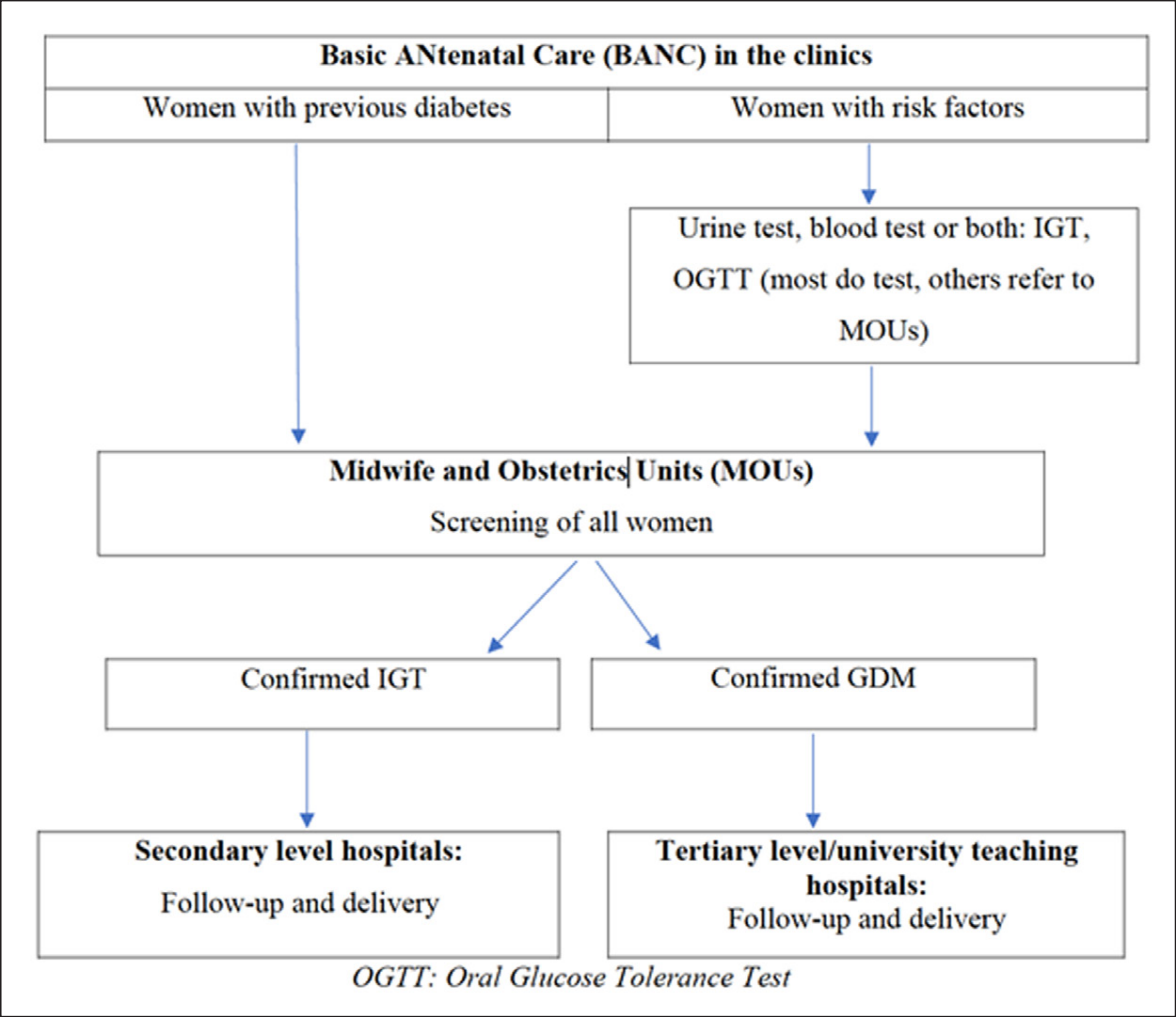
Process of diabetes screening during pregnancy in Cape Town.

The process of diabetes screening during pregnancy based on current guidelines in the local facilities in Cape Town is summarized in Figure 2. Referral starts from BANC, to Midwife and Obstetrics Units (MOU), to secondary level specialised maternity hospitals in case of impaired glucose tolerance (IGT – in which plasma glucose levels were above normal but below those defined as diabetes) [42,43], to tertiary hospitals (GSH or Tygerberg hospital depending on jurisdiction of the MOU) for cases meeting local criteria for GDM.

*“the procedure for screening, we’ve got a list of indications for doing Glucose Tolerance Test (GTT): family history of diabetes from her mother, her father or her siblings, BMI of 35 and above, history of big babies, persistent Glycosuria; for three consecutive visits. She has to come in the morning, fasting, her last meal the previous night around 10 o’clock. So, when she comes, we do the prick. If the sugar is 7 and above, we don’t continue, but if it is less than 7, we take the fasting blood and we give her 75 grams of glucose, and we take the second blood after two hours. So, they come after one week for the results. If it’s an IGT, we refer to Mowbray not Groote Schuur, but if it’s GDM, then we refer to Groote Schuur”*. **HCP 2**.

## Barriers to GDM screening in PHC

Respondents identified several barriers to screening. First of all, socio-economic factors impede the timely access of many women to BANC and to GDM screening. Secondly, some may be diagnosed late when symptoms or consequences of GDM are already present; this becomes a reason for immediate referral to the hospital for follow-up. Thirdly, there is no way to identify and screen some women as they do not attend ANC at the clinics at all. Fourthly, as many women, like other patients, do not know or suspect that they might have diabetes, they do not proactively seek any screening during their pregnancy or clinic visits, which is why the provincial guidelines mandate that screening be initiated by the provider. Finally, lack of time due to work overload, shortage or ineffective utilisation of key equipment and other resources for GDM screening, and poor communication between facilities, were also included among other documented health system issues preventing consistent GDM screening in PHC.

*“At the moment I think we have got one glucometer in the whole clinic, you understand? Sometimes we don’t know where it is and it is difficult to find it, you see…”*. **HCP 2**.

### Effective antenatal referral procedures but lack of follow-up after delivery

Themes in this category were appraised in the light of BCW’s intervention functions or middle layer [31], regarding services offered to women diagnosed with GDM while attending a diabetic clinic at hospital for follow-up and delivery.

### On-site integrated hospital services

Upon arrival at the respective hospital to which they are referred, women with GDM benefit from hospital level integrated care under the coordination of the diabetic clinic of the maternity department. Integrated services at referral hospitals include regular blood glucose monitoring, investigations for other health problems, medical care for GDM and other health problems beyond GDM, as well as diet and lifestyle change interventions.

*“We all have our specialities, so the registrar that would be looking after the patient is somebody that is rotated through the whole block, so they’ve seen cardiac, they’ve seen eclamptic patients, they’ve done diabetes; but if there is a specific problem, then we are in the fortunate position where we have the resources where we can get infectious disease people out, instead of struggling with that, or we can get the endocrinologist out, and say listen, we have now hit a wall, how do we go forward, but that is within our setting”*. **HCP 3**.

Counselling sessions regarding lifestyle changes to deal with diabetes and its devastating consequences were said to be routinely scheduled but not integrated within the services offered in the diabetic clinic of GSH’s obstetrics unit. As noted above, respondents reported that when counselling sessions were offered, crowded clinic conditions and lack of privacy decreased the effectiveness of sessions.

### Socio-economic barriers to challenges to healthy antenatal and postnatal initiatives

Women diagnosed with GDM at primary care and referred to tertiary hospital (GSH) (N = 35) discussed barriers they faced in their long road to care from families/communities, local facilities and up to referral hospitals, with many visits both during and after pregnancy. For many women the transport costs to attend care and the extra cost of healthy food contributed to depleting their already constrained economic resource.

*“…The diet food is actually very expensive compared to junk food. So, when I had to change, it was actually very hard, because I now have to spend much on my budget when it comes to my groceries, because of my diet and other food stuff for my boyfriend who is not diabetic”*. **Participant 3 in FGD 4**.

### Confusion or little knowledge about GDM and lifestyle changes

Apart from the socio-economic issues that women have to deal with in their daily lives, many have shown confusion or limited understanding of what GDM is and the behavioural/lifestyle changes required to manage GDM and prevent or delay future T2DM for themselves and long-term metabolic problems for their babies. Some women could not explain clearly what GDM was or why a particular treatment was prescribed to them while others struggled to name GDM consequences for themselves and their babies.

*“I also think GDM is when you are diabetic, they find out when you are pregnant, and then it’s not going to be seen after birth, but I was thinking like that before, but I have never actually known…that’s an impression, but I’m just assuming, I’m not sure”*. **Participant 1 in FGD 4**.

Women’s understanding about GDM as discussed in the FGDs was compared to the results from exit interview questionnaires. Despite the time they spent throughout the diagnosis and referral process at lower levels of health care, and after attending the diabetic clinic at GSH for their GDM care many times, only 43% reported having received advice about all four recommended actions (improve diet, reduce sugar intake, physical exercise and regularly take prescribed medication) to improve their GDM and prevent T2DM. However, women reported being satisfied with the information they had received, despite this lack of alignment with recommendations. Only half (51%) of the respondents were aware of the importance of reducing sugar intake, while 69% recalled being advised to exercise, 86% to improve their diet and 83% to take pills regularly. The contrast of improving diet (86%) and reducing sugar intake (51%) suggests incomplete and/or ineffective lifestyle change education.

**Figure 3:**
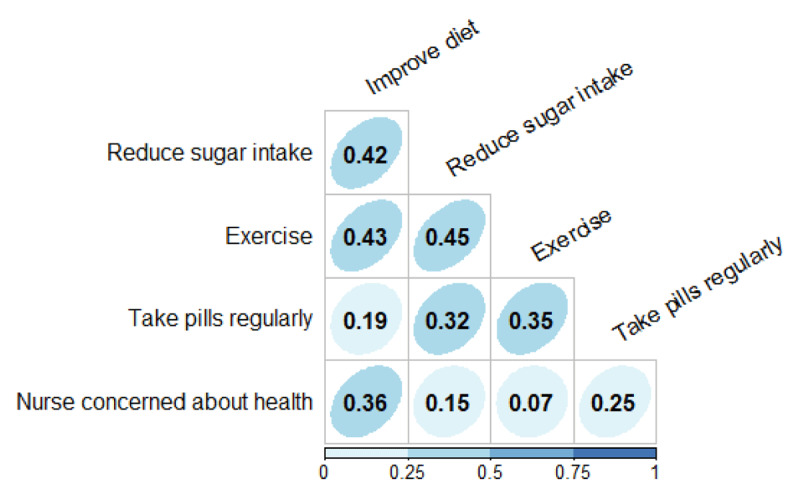
Multiple correlation between advices received by patients and their views on nurses’ interest in their health* * All variables have two categories “Yes” vs. “No”, except variable Nurse concerned about health whose two categories are “No concerned/Somewhat concerned” vs. “Appropriately concerned”.

Table 5 shows that, in contrast to the barriers reported by respondents regarding selective and late screening practices at primary care level, 94% kept their appointments at the referral hospital (GSH), 49% had already been tested in the morning before the FGDs were conducted, and 77% felt that nurses were interested or concerned about their health. Women who felt that nurses have empathy and time for them easily engaged with the nurses to ask about their GDM and general health, trusting their advice to change their lifestyle during pregnancy and postpartum to prevent or delay T2DM onset. Most women had their appointments every week (43%) or every 2 weeks (34%) with 86% reporting having received all their medications and not facing any stock-out. The multiple correlations between the advice that women with GDM received and their view on whether nurses were interested or concerned about their health, generally established a negligible, weak or moderate association as none reached 0.5. (Figure 3).

**Table 5:** Descriptive statistics.


FACTOR	LEVEL	VALUE

N (sample size)		35

Age (in years)	mean (SD)	33.7 (4.6)

	median (IQR)	34.0 (30.0, 37.0)

How long have you been attending diabetic clinic for your GDM care? (in days)	mean (SD)	106.9 (52.3)

	median (IQR)	120.0 (90.0, 120.0)

OGTT or blood glucose measured today?	Yes	17 (49%)

	No	9 (26%)

	Missing value	9 (26%)

Receive a SMS or a phone call to come to clinic?	Yes	1 (3%)

	No	33 (94%)

	Missing value	1 (3%)

Advices to reduce sugar intake?	Yes	18 (51%)

	No	17 (49%)

Advices to exercise?	Yes	24 (69%)

	No	11 (31%)

Advices to take my pills regularly?	Yes	29 (83%)

	No	6 (17%)

Advices to improve my diet?	Yes	30 (86%)

	No	5 (14%)

Number of advices received	One	8 (23%)

	Two	3 (9%)

	Three	9 (26%)

	Four	15 (43%)

The nurse was interested/concerned about your health?	No concerned	2 (6%)

	Somewhat concerned	6 (17%)

	Appropriately concerned	27 (77%)

Is there any medication that the nurse should have given you, but it is out of stock?	Yes	4 (11%)

	No	30 (86%)

	Missing value	1 (3%)

When is your return date?	1 Week	15 (43%)

	2 Weeks	12 (34%)

	1 Month	5 (14%)

	2 Months	1 (3%)

	Other	2 (6%)


**IQR:** Interquartile range; **SD:** Standard deviation.

### Poor communication and non-existent plans for postnatal follow-up

Even though integrated services including ANC and GDM were generally appreciated at referral hospitals, there was no follow-up for women and their babies after delivery. When women and their babies were back in their community after delivery, it was reported that they were seen at their local facilities exclusively for babies’ check-up and immunisation and that women did not have access to any specific programme that provided follow-up. Upon discharge, the details about their health were written up in their antenatal record (Road to Health booklet), but according to women and HCPs, the hospital does not consistently give or send a referral letter, nor call or communicate in other ways with local clinics regarding postnatal care. This is despite the guidelines indicating that a referral letter recommending a 6 week postpartum OGTT and follow up at a local clinic should always be handed to women at discharge. Both KIs and HCPs reported that, once back at the clinics, the women who did receive follow-up letters and medication from hospital tend to focus on their babies and forget or ignore to look after their own health. The few women who had approached the nurses at the primary care clinic regarding their postnatal check-up reported that they had not been successful in getting screened for diabetes.

*“The maternity sisters do not communicate with the local clinic sister for follow-up on these clients about medication after delivery and then we don’t know. So, maybe they got letters from hospital that you must follow up at this clinic to get your medication that is going to control you but mothers don’t follow up, as I have noted, they don’t follow up, they only focus on the baby after delivery,…… But if there is a problem, then the doctor prescribes when discharging them but they will never mention it to us at the clinic…And then, if they are with the person who didn’t see them when pregnant, you won’t know if the client had a problem with the glucose”*. **HCP 3**.

### IINDIAGO, an intervention with potential to bridge the gaps in postnatal follow-up

The BCW’s sources of behaviour or inner layer [31] was used to map and interpret failures in postnatal follow-up for mothers with previous GDM and their babies, a problem identified by all KI and HCPs approached for this research. This reported gap in postnatal care for women with GDM was also seen as an implementation vacuum that the newly approved WC postnatal policy aimed to solve [44] but respondents considered that the policy fell short in terms of follow-up for women who had GDM. The ongoing IINDIAGO study that aims to integrate post-partum follow up for women post-GDM into PHC was presented to respondents at the end of the interview, in order to explore the perceived relevance and feasibility of such an intervention. The idea was welcomed and seen as feasible by all respondents including women.

*Well, the issue about IINDIAGO is that you are actually addressing the exact problem. I think it’s feasible, given the right funding. I have no doubt, you know, things like weight reduction and proper dietary counselling etc. can prevent the development of Type 2*. **KI 3**.

### Appreciation of CHWs involvement in community based T2DM prevention intervention

The BCW’s sources of behaviour or inner layer [31] was applied to help understand the changes that need to take place in the community in order to prevent or delay T2DM. Since the overwhelmed clinics do not intervene much, if at all, in T2DM prevention efforts, CHWs were considered to be the best-placed health workers to successfully contribute to implementation of activities in the family and community. CHWs in South Africa have greatly assisted [45,46] in other community-based interventions to improve health, principally in the areas of maternal and child health and HIV care. Existing policy also gives them a role to play in non-communicable diseases (NCDs) [47]. All participants (KIs, HCPs, Women) commended CHWs and suggested that they get involved in T2DM prevention once trained and working under clinic supervision. KIs suggested that their involvement could bring some clinical services like NCDs screening, counselling, health education, and implementation of specific preventive measures to the patients and family members within communities.

Reflecting on their experiences with HIV and tuberculosis, the CHWs who participated in this study responded positively to the idea of getting involved in such innovative and integrated approach towards T2DM prevention for women who had GDM. CHWs explained how their visits to the families within community are more inclusive and go beyond the single patient they are scheduled to visit, covering a range of health problems of all present family members. Equipped with their household charts, they reported that they conduct a complete surveillance of the family and refer family members with particular health problems to the right health facility for further diagnosis and care. CHWs emphasised their visiting and educating roles would align well with the tasks they would handle in T2DM prevention efforts. These positive comments were made despite reporting challenges they face in their daily activities like limited training; low and irregular payments; very busy clinics that sometimes fail to follow up the patients they refer to them. CHWs expressed commitment to their cause and engagement in their mission within the community.

*“Me, I love the job that I am doing because I don’t have a problem with people, and I can convince them but if someone is not doing well, I report her to the supervisor who will then intervene”*. **CHW participant 3, FGD 1**.

## Discussion

In the face of increasing GDM prevalence in Africa [48] and despite calls for universal screening, the guidelines in most countries recommend selective screening to diagnose and manage GDM and its sequelae [49,50]. Risk factor-based screening has been the main approach adopted in South Africa. Even though the current GDM screening guidelines in South Africa now meet international standards, respecting the value thresholds as recently discussed by Adam S. and Rheeder [19], they are still ineffectively applied. The “Basic antenatal care (BANC) protocol” was identified as the main tool used for antenatal service provision in most of Cape Town clinics but is a complex guideline with many components [17]. Ultimately the decision to screen GDM or not lies with the nurses, in line with the facility plan rather than this complex protocol itself.

Documented challenges in GDM testing at primary care level were a sign but also a cause of poor screening practice. Universal screening of GDM cannot be successful if concurrent barriers are not addressed. These challenges to GDM screening in PHC include but are not limited to shortages of well-trained HCPs and ill-equipped clinics to test and deal with NCDs based on the available guidelines [51]. Multiple barriers impeding proper GDM screening and follow up post-GDM have been documented in other studies and this study’s findings corroborate many, including: weaknesses at different health system levels; poor understanding of postpartum GDM risks of T2DM development for both women and their babies; and various patient, community and health service level barriers for women when they are referred back into PHC for follow-up after delivery [15,52,53]. Our findings further suggest that the expertise and knowledge required of both nurses and women are insufficient to make a risk factor-based approach effective in South Africa.

It has never been easy for women to navigate health systems to access obstetric care in sub-Saharan Africa due to multiple individual and family socio-economic barriers such as low household income, illiteracy, lack of transport means and its cost, and cultural beliefs/practices, among others as reported in recent studies [54,55]. Despite these issues, women receive integrated and highly appreciated antenatal and perinatal care at the tertiary level. Women with GDM who participated in this study confirmed this. However, women who strived to protect their babies from the adverse effects of GDM feel relieved after delivery and this is reinforced after their glucose levels return to the normal range. Additionally, the lack of structured postnatal care for these women does not foster the implementation of T2DM prevention initiatives.

Our findings suggest that this could be at least partially mitigated with clear and consistent discussions about GDM and its long-term consequences for both women and their babies throughout ANC, perinatal and post-partum services. Health education may encourage these women to follow-up with postnatal testing and lifestyle change measures at the clinic and in the community. Referral hospitals must first communicate with the local facilities regarding follow-up for these women and, in return, the clinics need to continue surveillance and initiate integrated postnatal behavioural change interventions for T2DM prevention. Such interventions would be useful for other NCDs and broader health care needs beyond the immediate aim of dealing with IGT, T2DM or diabetes related health issues but to achieve this, nurses need appropriate training and more resources in the facilities.

The IINDIAGO project is exploring whether such postnatal follow-up could be linked to the babies’ immunisation, which normally starts soon after delivery and discharge from hospital. HCPs showed willingness to add this programme to their workload after receiving proper guidelines and adequate training on their side. Women also expressed support for this kind of intervention after discussing its dual benefits, for them and for their babies. Engaging policy makers to change guidelines on the one hand and appropriately train frontline healthcare workers including CHWs on the other has succeeded in other trials and interventions in PHC for the same populations. Here, Prevention of Mother-to-Child Transmission of HIV (PMTCT) which continues from ANC into postnatal care with lifelong services within the facility and in the community [56,57,58] could serve as a case study.

## Conclusion

Effective care of GDM and prevention or delay of T2DM requires a continuum of care from screening and diagnosis of GDM, to antenatal and intrapartum management, to post-partum follow up and prevention interventions. Despite policy support and guidelines promoting integrated care, implementation of GDM screening, delivery of counselling about GDM and T2DM, and post-partum follow up are suboptimal in Western Cape. Many women are diagnosed late in their pregnancy and postnatal follow-up is almost non-existent. An innovative strategy of integrating universal GDM screening in local health facilities with postnatal follow-up of these women and their babies in the community based PHC services is considered desirable and feasible by all participants in this study. Women, health providers, and experts added that this integration would work well if the resource and training constraints facing PHC as well as socio-economic barriers to women are addressed.

## Data Accessibility Statements

The datasets analysed during the current study are not publicly available to preserve participant anonymity.

## Additional File

The additional file for this article can be found as follows:

10.5334/ijic.5600.s1 Additional File. Mixed Methods Appraisal Tool (MMAT), version 2018.

## Figures and Tables

**Table:** 

ADA	American Diabetes Association
ANC	AnteNatal Care
ART	Anti-Retroviral Therapy
BANC	Basic ANtenatal Care
BCW	Behaviour Change Wheel
CHWs	Community Health Workers
DPSG	Diabetes Pregnancy Study Group
EASD	European Association for the Study of Diabetes
GDM	Gestational Diabetes Mellitus
GSH	Groote Schuur Hospital
HCPs	Health Care Providers
HIV	Human Immunodeficiency Virus
IADPSG	International Association of Diabetes and Pregnancy Study Groups
IGT	Impaired Glucose Tolerance
IINDIAGO	Integrated Intervention for Diabetes risk after Gestational diabetes
IQR	InterQuartile Range
KIs	Key Informants
LMICs	Low and Middle-Income Countries
MMAT	Mixed Methods Appraisal Tool
MOU	Midwife and Obstetrics Unit
NCDs	Non-communicable diseases
NDDG	National Diabetes Data Group
NIMART	Nurse -Initiated Management of AntiRetroviral Therapy
OGTT	Oral Glucose Tolerance Test
PHC	Primary Health Care
PICT	Provider-initiated counselling and testing
PMTCT	Prevention of Mother-To-Child Transmission
PNC	Post Natal Care
SA	South Africa
SD	Standard Deviation
SSA	Sub-Saharan Africa
T2DM	Type 2 Diabetes Mellitus
WC	Western Cape.
